# Prevention of upper aerodigestive tract cancer through active search strategies and use of equipped propaedeutics^☆^^☆☆^^☆☆☆^

**DOI:** 10.1016/j.bjorl.2019.01.002

**Published:** 2019-02-20

**Authors:** Francis Balduino Guimarães Santos, Fernando Danelon Leonhardt, Márcio Abrahão

**Affiliations:** a Universidade Estadual de Montes Claros (Unimontes), Curso de Medicina, Montes Claros, MG, Brazil; b Universidade Federal de São Paulo (Unifesp), Programa de Pós-Graduação em Otorrinolaringologia (PPG/ORL), São Paulo, SP, Brazil

**Keywords:** Upper aerodigestive tract neoplasia, Secondary prevention, Prevalence, Neoplasia do trato aerodigestivo superior, Prevenção secundária, Prevalência

## Abstract

**Introduction:**

Upper aerodigestive tract cancer is among the most frequent malignancies and has epidemiological importance worldwide. Most cases are already advanced at the diagnosis, with a strong negative impact on survival and high cost to the government. Campaigns directed against these cancers have often failed in Brazil.

**Objective:**

To evaluate the effectiveness of screening for upper aerodigestive tract cancers, using active search strategies and the use of equipped propaedeutics.

**Methods:**

A cross-sectional, prospective, descriptive, analytical and exploratory study, since its objectives are based on the visualization of cancerous lesions in a sample consisting of individuals with risk factors, aiming to expand the necessary knowledge for cancer detection, aiming at secondary prevention of the oral cavity, oropharynx, larynx and hypopharynx cancer.

**Results:**

A total of 16.7% of precancerous lesions and 0.5% of cancer lesions located in the upper aerodigestive tract were clinically visualized.

**Conclusion:**

The method was effective in the identification of precancerous lesions for the purpose of secondary prevention, but equally important against upper aerodigestive tract cancer, since in the present study the chance of finding the latter was increased by 22.7, showing it is an alternative for future campaigns against the disease.

## Resumo

**Introdução:**

O câncer do trato aerodigestivo superior configura-se entre os mais frequentes e apresenta-se com importância epidemiológica no mundo. A maior parte apresenta-se avançada ao diagnóstico, com forte impacto negativo na sobrevida e elevado custo ao erário. As campanhas feitas contra esses cânceres têm frequentemente falhado no Brasil.

**Objetivo:**

Avaliar a efetividade do rastreamento para lesões cancerizáveis do trato aerodigestivo superior com estratégias de busca ativa e uso da propedêutica armada.

**Método:**

Estudo transversal, prospectivo, descritivo, analítico e exploratório, uma vez que os seus objetivos se alicerçam na visualização das lesões cancerizáveis numa amostra composta por indivíduos com fatores de risco, de forma a aprofundar o conhecimento necessário para a sua detecção, visando à prevenção secundária do câncer da cavidade oral, orofaringe, laringe e hipofaringe.

**Resultado:**

Foram visualizadas clinicamente 16,7% lesões cancerizáveis e 0,5% cânceres de localização no trato aerodigestivo superior.

**Conclusão:**

O método mostrou-se efetivo na identificação de lesões cancerizáveis com intuito de prevenção secundária, mas igualmente importante contra o câncer do trato aerodigestivo superior, uma vez que neste presente estudo multiplicou-se a chance do encontro desse por 22,7; apresenta-se como opção para futuras campanhas contra a doença.

## Introduction

Because of the similarity in their natural history, cancers that affect the oral cavity, oropharynx, larynx, and hypopharynx are usually described as “squamous cell carcinoma of the upper aerodigestive tract (UADT)”, “head and neck cancer,” or, as reported in lay language as “cancer of the mouth and throat.” Even though they are different diseases and affect different anatomical regions, considering them together may be relevant in an epidemiological context and for the development of appropriate public health actions.^1^

UADT carcinoma is the sixth most prevalent type of cancer in the world with 9.2% of cases, and is globally responsible for 4.6% of cancer-related deaths.^2^ In Brazil, for the 2018/19 period, the National Cancer Institute (INCA)^3^ estimated 22,370 new cases of UADT carcinoma (C00-10, C32), of which 17,590 cases were found in males and 4780 in females, corresponding respectively to the 3rd most prevalent cancer in males and the 12th in females. Still according to INCA, the cases of UADT carcinoma in both genders account for an estimated risk of 21.51 new cases per 100,000 inhabitants. This is a serious public health problem, not only because of its incidence, but because the cancer stage is advanced in 75% to 80% of the cases at the time of diagnosis in Brazil, with a mean mortality rate of 46% in 5 years, which has remained unchanged in the last two to three decades.^4^, ^5^, ^6^

The main risk factors^7^ for UADT carcinoma include the smoking habit, alcohol consumption, sun exposure for lip cancer and Human Papilloma Virus (HPV), especially in the oropharynx. This is an asymptomatic or oligosymptomatic disease in its initial course, often presenting as the main pre-cancerous lesions (PCLs) or precursor lesions erythroplakia, erythroleukoplakia and leukoplakia.^7^, ^8^

Several barriers to an early diagnosis have been described, which seem to be related to the population's lack of knowledge about risk factors, poor social support, low levels of schooling and *per capita* income, demographic/geographical aspects, and poor access to health services.^9^, ^10^ Considering the Brazilian reality, there is no possibility to act on all the above mentioned factors, and for this reason it has been suggested that the early finding of a Pre-Cancerous Lesion (PCL) in the Upper Aerodigestive Tract (UADT) is the most effective form of successful prevention and clinical intervention,^11^ which would be possible with prevention campaigns.

However, prevention campaigns against cancer of the oral cavity and larynx initiated in Brazil are mostly sporadic, carried out in individuals with/without risk factors, with ages outside the age group, with higher prevalence due to the broad appeal in the community and inattention to pre-cancerous lesions. In part, the target population of these campaigns do not exclusively consist of the at-risk group, partly explaining the low number of cancer detection cases.^12^ However, one should not disregard the element of public awareness and information against the several types of cancer, but a better understanding of the biological and epidemiological processes for UADT carcinoma prevention is necessary.

Unlike conventional prevention campaigns, one can attract the individuals who fit the high-risk profile, inviting them to medical care through an active search.^13^ This strategy was used in Kerala (India) in a campaign against cancer of the oral cavity, where screening results were considered satisfactory.^14^ Subsequently, a strategy focused on the visualization and therapeutic approach of PCLs may be feasible in reducing UADT carcinoma incidence and mortality.^15^ Consistent with the literature, equipped propaedeutics through video-laryngoscopy (VL) or Video-nasolaryngoscopy (VNL) is a safe and effective technique to visualize lesions or alterations in the UADT epithelium.^16^

## Methods

This was a cross-sectional, prospective, descriptive, analytical and exploratory study, an interinstitutional research project, Plataforma Brasil – CAAE: 28081014.2.1001.5505, REC of Universidade Federal de São Paulo/UNIFESP – Opinion No. 618.107 and REC of Universidade Estadual de Montes Claros/UNIMONTES – Opinion No. 006/2015, which aimed to evaluate the effectiveness of screening for PCLs in the UADT through the active search strategy concomitant to equipped propeadeutics in individuals with risk factors for UADT carcinoma. It also aimed at measuring the magnitude of the associations between PCLs and the studied variables.

The lack of knowledge on the prevalence (*P*) of UADT carcinoma PCLs in our country does not allow us to infer values, and, thus, the statistical rule for sample inference was^17^: *n*_0_ = *z*^2^ × *P*(1 − *P*)/*E*^2^, where: *p* = 0.5; *z*^2^ if the 95% IC = 1.96 and *E*^2^ = sample error of 4% → *n*_0_ = 600. The sample consisted of 603 individuals living in the municipality of Montes Claros, state of Minas Gerais, Brazil, of both genders, aged ≥40 years, who were smokers and/or alcoholics. These were screened by the Family Health Strategy in the several territories in a rotation system and sent to the referral ambulatory – active search, where they were submitted to oroscopy and to the equipped propeadeutics by a single specialist through the conventional 8 mm/70° rigid telescope (VL) or adult flexible nasofibroscope (VNL) in those who were intolerant to the first.

The studied variables were distributed as sociodemographic (age, gender, marital status, level of schooling, professional status), clinical (smoking and alcohol abuse classifications, presence of PCL) and clinicopathological variables (macroscopic PCL presentation and its anatomical location).

A heavy smoker was the one who consumed ≥40 packets/year^18^ and a heavy drinker was the one who consumed ≥5 units of alcohol/day (1 unit = 8 g of alcohol).^19^

Leukoplakia, lichen planus, actinic cheilitis, erythroplakia, submucosal fibrosis and verrucous lesions of the oropharynx were considered macroscopically as PCLs.^20^, ^21^ PCLs suspected of being cancer were submitted to biopsy and histopathological study, considering the need for immediate treatment.

The statistical analysis was performed using the SPSS V. 21® program. Initially, a descriptive analysis of the data was performed, followed by non-parametric tests with bivariate analysis (*X*^2^), bivariate logistic regression model, and the adjusted and hierarchical multiple logistic analysis of the data (OR, 95% CI). Significance level was set at 5% (*p* = 0.05).

## Results

The inclusion criteria were met by 603 individuals and, considering the sociodemographic variables (Table 1), the mean age was 55.3 years ± 11.51 (Min: 40/Max: 90) with a prevalence of the male gender, consisting of 334 male individuals (55.4%); 306 married individuals (50.7%); level of schooling ≤8th grade in 419 (69.5%); retired, 138 (22.9%) and homemakers/similar activities in 104 (17.2%).

**Table 1:** Frequencies and percentages of the sample sociodemographic variables. Bivariate analysis by chi-square between PCLs according to sociodemographic variables.

Sociodemographic variables	Sample^a^ *n* (%)	Absence of PCLs *n* (%)	Presence of PCLs *n* (%)	*X*^2^
*n (%)*	603 (100)	499 (82.8)	101 (16.7)	

*Age*^b^				0.58
40 to 59 years	414 (68.7)	347 (69.1)	67 (66.3)	
≥60 years (elderly)	189 (31.3)	155 (30.9)	34 (33.7)	

*Gender*				0.05^a^
♀	269 (44.6)	231 (46.3)	36 (35.6)	
♂	334 (55.4)	268 (53.7)	65 (64.4)	

*Marital status*				0.30
Married	306 (50.7)	245 (48.8)	61 (60.4)	
Single/widowed/separated	297 (49.3)	257 (51.2)	40 (39.6)	

*Level of schooling*				0.25
≤8th grade	419 (69.5)	344 (68.5)	75 (74.3)	
≥High school	184 (30.5)	158 (31.5)	26 (25.7)	

*Profession*^c^				–
Retired	138 (22.9)	108 (21.6)	28 (27.7)	
Homemaker	104 (17.2)	89 (17.8)	15 (14.9)	
Salesclerk	78 (12.9)	62 (12.4)	16 (12.8)	
Farmer	67 (11.1)	57(11.4)	9 (8.9)	
Construction worker	54 (9.0)	40 (8.0)	14 (13.9)	
Mechanic-machine operator	36 (6.0)	30 (6.0)	6 (5.9)	
Health care worker	24 (4.0)	23 (4.6)	1 (1.0)	
Others sectors	102 (16.9)	90 (18.0)	12 (11.9)	

a Cancer cases are included in the sample.

b World Health Organization.^22^

c Brazilian Classification of Occupations.^23^

PCLs were identified visually in 101 individuals (16.7%), with 65 cases in males (64.4%); in their 4th and 6th decades of life in 67 (66.3%) and with level of schooling ≤8th grade in 75 (74.3%). Through the bivariate analysis, only the male gender (*p* = 0.05) was statistically associated with the PCLs.

Regarding the clinical variables (Table 2), all the individuals in the sample were submitted to oroscopy, of which 525 to armed propaedeutics through VL (87.1%) and 78 through VNL (12.9%).

**Table 2:** Frequencies and percentages of the sample clinical variables. Bivariate analysis by Chi-square between PCLs according to the clinical variables.

Clinical variables		Sample *n* (%)	Absence of PCLs *n* (%)	Presence of PCLs *n* (%)	*X*^2^
*n* (%)		603 (100)	499 (82.8)	101 (16.7)	
Smoking habit	Present	547 (90.7)	454 (90.4)	93 (92.1)	0.60
	Absent	56 (9.3)	48 (9.6)	8 (7.9)	
Smoking classification^a^	Mild/moderate	146 (31.3)	131 (34.4)	15 (17.6)	0.003^a^
	Heavy	320 (68.7)	250 (65.6)	70 (82.4)	
Alcohol consumption habit	Present	483 (80.1)	397 (79.1)	86 (85.1)	0.16
	Absent	120 (19.9)	105 (20.9)	15 (14.9)	
Alcohol consumption classification^b^	Mild/moderate	268 (68.9)	221 (69.1)	47 (68.1)	0.88
	Heavy	121 (31.1)	99 (30.9)	22 (31.9)	

The former smoker^a^ and former alcohol abuser are excluded^b^.

Regarding the assessed habits that were deleterious to health, smoking was observed in 547 (90.7%) individuals and alcohol abuse in 483 (80.1%). Among the former and disregarding the ex-smoker, 320 (68.7%) were classified as heavy smokers and among the latter, and disregarding the ex-alcohol abuser, 121 (31.1%) were classified as heavy drinkers.

Among the individuals with PCLs, smoking was observed in 93 individuals (92.1%) and alcohol abuse in 86 (85.1%). Through the bivariate analysis, only the heavy smokers (*p* = 0.003) showed a statistically significant association with PCLs.

Considering the clinicopathological variables (Table 3), PCLs were distributed as leukoplakia in 92 (91.1%), erythroplakia in 7 (6.9%) and verrucous lesion in 2 cases (2.0%). As for the anatomical site distribution, 65 (64.4%) of the PCLs were found in the oral cavity, 29 (28.7%) in the larynx and 7 (6.9%) in the oropharynx. No PCL was identified in the hypopharynx.

**Table 3:** Frequencies and percentages of clinicopathological variables (PCLs) according to the anatomical location and clinical presentation.

Clinicopathological variables	Oral cavity *n* (%)	Oropharynx *n* (%)	Larynx *n* (%)	Total *n* (%)
*Leukoplasia*	65 (64.4)	7 (6.9)	29 (28.7)	101 (100)
	54 (83.1)	5 (71.4)	26 (89.0)	85 (84.1)
*Precancerous lesions*				
Erythroplakia^a^	4 (6.2)	0 (0)	3 (10.3)	7 (6.9)
Actinic cheilitis^b^	5 (7.7)	0 (0)	0 (0)	5 (5.0)
Lichen Plano^b^	2 (3.1)	0 (0)	0 (0)	2 (2.0)
Verrucous lesion	0 (0)	2 (28.6)	0 (0)	2 (2.0)

a Includes leukoerythroplakia.

b Visualized as leukoplakia.

Through the bivariate logistic regression (crude) followed by adjusted and hierarchized multiple regression, only the heavy smoker (*p* = 0.003) showed a statistically significant association with the PCLs (Table 4).

**Table 4:** Association between PCLs according to sociodemographic and clinical variables estimated by the bivariate logistic regression model and by the adjusted and hierarchical multiple analysis. Odds ratio (OR) with 95% confidence interval (95% CI).

Variables		Crude OR (95% CI)	*p*	Adjusted OR (95% CI)	*p*
*Level I*					
Socio-demographic	Gender	1.56 (0.45–0.23)	0.048	1.28 (0.30–0.27)	0.27

*Level II*					
Clinical	Alcohol consumption	1.52 (0.42–0.30)	0.17	1.51 (0.41–0.35)	0.24
	Heavy smoker	2.45 (0.89–0.30)	0.003	2.60 (0.96–0.32)	0.003^a^

Only the variables with *p* ≤ 0.20 in the crude OR followed the adjusted and hierarchical OR.

a OR *p* ≤ 0.05.

Finally, the 3 (0.5%), squamous cell carcinomas in the sample were visualized, biopsied and staged (TNM/2017),^24^ of which one was in the oral cavity “In Situ”, another in the glottic larynx, Stage I and the third in the hypopharynx, Stage III.

## Discussion

In this context of an active search concomitant to the use of armed propaedeutics in individuals with risk factors, an epidemiological profile was obtained in the sample that was similar to the patients with UADT carcinoma, consisting mostly of males (55.4%), with a mean age of approximately 55 years, with low level of schooling (69.5%); with smoking habits (90.7%) and alcohol abusers (80.1%).^25^

The PLCs were visualized in 16.7% of the sample, spread in the oral cavity, oropharynx, larynx, with none being visualized in the hypopharynx. They were statistically associated with the male gender in 64.4% (*p* = 0.05), and to the heavy smoker in 82.4% (*p* = 0.003), with the adjusted and hierarchical multiple analysis (*p* = 0.003) showing a statistically significant association with the latter, with a 2.6-fold higher risk. These findings are consistent with the literature, since men are more exposed to risk factors for UADT carcinoma when compared to women, with smoking alone being the most important risk factor.^25^

For this study, the oral cavity was the most prevalent anatomical site, with 10.8% of PCLs, and their visualization depended only on oroscopy and varied within the margins described in the literature. The rate of detectable PCLs in the oral cavity in the overall population ranges from 1% to 5%,^26^ but for the male gender, smokers and those aged >40 years, it can reach rates of 9% to 12.7%.^27^

The visualization of PCLs in the oral cavity occurred more frequently and may be due, in part, to extensive and prolonged contact with the carcinogenic agents present in tobacco and alcoholic beverages, especially strong distilled liquor.^28^ The visualization of PCLs in the larynx corresponded to 4.8%, being the second anatomic site in prevalence and depended on the armed propaedeutics. The rate of detectable PCLs in the larynx in the Brazilian population is still uncertain, but microsurgical findings in individuals with previous laryngeal epithelial alterations ranged from 7.5% to 18.5%.^29^

In the oropharynx, the visualization of PCLs occurred in 1.2%, and depended partially on the armed propaedeutics for the adequate evaluation of the base of the tongue. Different from the oral cavity, the epidemiological descriptions of PCLs in the oropharynx, larynx and hypopharynx are scarce in the literature, thus preventing accurate comparisons.^30^

Regarding the clinicopathological presentation, leukoplakia accounted for 91.1% of PCLs (including lichen planus and actinic cheilitis), with a higher occurrence in the oral cavity in almost 2/3 of the cases, followed by the larynx with 28.3% and oropharynx with 5.4%. In the oropharynx, in addition to leukoplakia, verrucous lesions were visualized (2.0%), which can be investigated through immunohistochemistry for HPV-16 infection. Erythroplakia occurred in 6.9% of the oral cavity and larynx, with a preference for the first. Although less frequent, erythroplakia in the UADT has a high carcinogenic potential and deserves a decisive therapeutic approach.

There are current recommendations for follow-up of the individual with PCLs in the UADT through armed propaedeutics, with the author stating that “... these are individuals who deserve to be watched permanently and in which the use of imaging techniques more than doubles the rate of detectable malignant lesions”.^31^ Another crucial point for the secondary prevention of UADT is based on the evaluation of PCLs regarding their carcinogenic potential through pathological analysis and definition of degrees of dysplasia, which is not well defined in a considerable number of prevention campaigns.

Currently in Brazil, many prevention campaigns for UADT carcinoma have a wide appeal to the population and a major focus on tertiary prevention, mobilizing human resources, bringing higher costs to the public sector or to the proponent institutions and showing low efficacy, inducing an author to say that “... it is necessary to evaluate the advisability of carrying out the campaign in the next years”.^12^ Another example is based on the oral cancer prevention campaign called “Open your Mouth for Health” in the city of São Bernardo do Campo/São Paulo, of which results showed that the population at risk for the development of oral cavity cancer. “is not being effectively reached by the prevention campaign, a fact that is probably also occurring in most campaigns in Brazilian cities.”^32^

Although this study aimed at visualizing PCLs in the UADT, 3 (0.5%) UADT carcinomas were diagnosed, of which 2 (66.6%) were classified as being in the initial stages.

The finding of cancer in this study was very relevant, since it significantly exceeded the expected proportion of UADT carcinoma in 22.7 times and more, when considering the Brazilian population with 21.51 new cases per 100,000 inhabitants or 0.022%; this presumably caused by the selection of the individuals at risk and by the use of the armed propaedeutics, which extended the anatomical areas to be evaluated. This becomes interesting, since large-scale screening programs remain to be established regionally or globally, considering the scarcity of health resources in our country.^33^

Considering the number of assessed individuals, we believe it is possible to generalize the obtained results to other regions of our countryside that have a similar population density and socioeconomic and cultural characteristics.

## Conclusion

The assessed method was valid not only for the identification of PCLs (16.7%) in the secondary prevention of UADT carcinoma, but equally important in identifying it at high rates when compared to the traditional campaigns (22.7-fold higher), demonstrating that it is a new alternative to be explored.

## Conflicts of interest

The authors declare no conflicts of interest.
